# Convergent and Divergent fMRI Responses in Children and Adults to Increasing Language Production Demands

**DOI:** 10.1093/cercor/bhu120

**Published:** 2014-06-06

**Authors:** Saloni Krishnan, Robert Leech, Evelyne Mercure, Sarah Lloyd-Fox, Frederic Dick

**Affiliations:** 1 Birkbeck-UCL Centre for NeuroImaging, London, UK; 2 Centre for Brain and Cognitive Development, Birkbeck, University of London, London, UK; 3 Department of Neurosciences and Mental Health, Imperial College London, London, UK; 4 Institute of Cognitive Neuroscience, UCL, London, UK

**Keywords:** development, fMRI, language production, task difficulty, word generation

## Abstract

In adults, patterns of neural activation associated with perhaps the most basic language skill—overt object naming—are extensively modulated by the psycholinguistic and visual complexity of the stimuli. Do children's brains react similarly when confronted with increasing processing demands, or they solve this problem in a different way? Here we scanned 37 children aged 7–13 and 19 young adults who performed a well-normed picture-naming task with 3 levels of difficulty. While neural organization for naming was largely similar in childhood and adulthood, adults had greater activation in all naming conditions over inferior temporal gyri and superior temporal gyri/supramarginal gyri. Manipulating naming complexity affected adults and children quite differently: neural activation, especially over the dorsolateral prefrontal cortex, showed complexity-dependent increases in adults, but complexity-dependent decreases in children. These represent fundamentally different responses to the linguistic and conceptual challenges of a simple naming task that makes no demands on literacy or metalinguistics. We discuss how these neural differences might result from different cognitive strategies used by adults and children during lexical retrieval/production as well as developmental changes in brain structure and functional connectivity.

## Introduction

Language takes a long time to master. Even in the school years, children are still converging on the representational structures and processing strategies that adults typically use in everyday language comprehension and production (Berger et al. 1996; Metsala 1997; Brooks and MacWhinney 2000). As in other domains like visuo-spatial cognition (Akshoomoff and Stiles 1995a; 1995b; Stiles, Akshoomoff and Haist 2013), developmental differences in language skills will often come to light as the difficulty or complexity of the linguistic material or task demands increase (Simpson and Foster 1986; Cycowicz et al. 1997; Brooks and MacWhinney 2000; Leech et al. 2007). As shown in several functional magnetic resonance imaging (fMRI) studies, varying the complexity or focus of a cognitive task can also reveal underlying developmental shifts in functional organization, even when task performance is very similar across the age range (Cohen Kadosh et al. 2010, 2011, 2013). In order to understand how a child's brain organizes itself to best solve the problem of producing language—and how this organization differs from the “mature” brain—it may be particularly useful to see what happens when the language production task becomes increasingly demanding.

Picture naming is an ideal task in this regard as it is a ubiquitous and spontaneously occurring communicative act across the lifespan—but one that can vary considerably in its complexity. Toddlers show off their latest lexical acquisitions while looking at picture books and family photos. School children and college students learn to identify and name increasingly esoteric objects in their English, biology, and anatomy classes. Throughout adulthood, we name representations of objects that we see in order to understand, categorize, classify, and to communicate what we are experiencing—and what we want—to our fellow interlocutors.

The visual, cognitive, and linguistic factors affecting picture-naming performance are very well characterized across the lifespan (Berman et al. 1989; Cycowicz et al. 1997; D'Amico, Devescovi, and Bates 2001; Iyer et al. 2001) and across languages (Bates et al. 2003; Szekelky et al. 2003). Neuroimaging studies have shown how adults’ brain activation is modulated by subtle manipulations of word frequency, semantic content, and visual complexity (Indefrey and Levelt 2004; Price et al. 2005; Graves et al. 2007; Wilson et al. 2009; Indefrey 2011; Price 2012).

Somewhat surprisingly given its ecological validity as a naturalistic task that is relatively free of metalinguistic task demands, the neural development of picture or object naming has been little studied using fMRI. To our knowledge, only 3 fMRI studies have been carried out that involve children naming an object in some way. Grande et al. (2011) studied German-speaking dyslexic and typically reading children, who named pictures of or read low- and high-frequency words. No activation differences between dyslexic and typically reading children were observed in the naming task. However, for both reading and naming, an overall effect of word frequency was observed in the left inferior frontal gyrus (IFG). De Guibert et al. (2010) studied 18 children using novel tasks involving covert naming, but did not explore developmental change. Turkeltaub et al. (2008) describe changes in activation for object naming over development but restrict their analysis to the ventral stream.

Much of what we do know about the development of language production is based on a series of important developmental studies using well-characterized adult neuropsychological and/or fMRI language tasks that do not involve picture naming. These include simple word repetition to an auditory cue (Church et al. 2008), overt word reading (Schlaggar and McCandliss 2007; Church et al. 2008; Heim et al. 2010; Grande et al. 2011), word generation to a category (Gaillard et al. 2000, 2003) or more metalinguistic tasks such as verb, rhyme, or antonym generation to a read or heard cue word (Holland et al. 2001; Schlaggar et al. 2002; Schapiro et al. 2004; Brown et al. 2005; Szaflarski, Schmithorst et al. 2006).

In a seminal study, Brown et al. (2005) studied a large group of children and adults performing overt word generation tasks (rhyme, verb, and opposite generation). By comparing adults and children with similar accuracy/reaction times on these tasks, they identified regions where age-related decreases (bilateral medial frontal, parietal, occipitotemporal and cingulate cortex) and age-related increases in activity (left lateral and medial frontal regions) were observed. In contrast, by comparing adults and children whose performance differed, changes were noted over the right frontal cortex, medial parietal cortex, posterior cingulate, and occipital cortices bilaterally. These results were suggestive of increased activity in newly recruited regions such as frontal cortex over development, and increased specialization of activity in earlier processing regions such as extrastriate cortex. In a related study, Church et al. (2008) compared 2 language production tasks over development (reading and repeating words aloud). Age-related decreases were observed over temporal and occipital cortices. However, activation was modulated by task. In the reading task, adults showed decreased activity in regions associated with phonological processes, such as the angular and supramarginal gyri.

Other developmental fMRI studies of language production have focused on identifying patterns of language lateralization in individuals (Gaillard et al. 2004; Berl et al. 2014a), over tasks (Bookheimer et al. 2000; de Guibert et al. 2010; Lidzba et al. 2011) and over response modalities (Croft et al. 2013). In right-handed adults, left-lateralization is considered a hallmark of language organization. By and large, developmental studies indicate that while most school-age children show left-lateralized responses, there are individual and task-dependent differences. Age-related changes in lateralization have also been described: for example, Szaflarski, Holland et al. (2006) showed that neural activation for a covert verb generation task became increasingly left lateralized between childhood and adolescence. In particular, age-related changes within so-called Broca's area have been a focus of many developmental language studies (reviewed in Berl, Mayo et al. 2014). However, the conclusions that can be drawn appear to depend on the age range of the sample in question, as well as specific task demands. In general, studies that have used verbal fluency or categorization tasks tend to show age-related increases in activation over the left IFG (Holland et al. 2001), whereas studies using semantic association tasks tend to evoke differences over the right IFG (Booth et al. 2003; Chou et al. 2006). Yet other studies report that activation changes and increasing left-lateralization in the IFG are related to age (Berl, Mayo et al. 2014), performance (Blumenfeld et al. 2006; Bach et al. 2010) or socioeconomic status (Raizada et al. 2008).

### The Present Study

These developmental studies of language production have given us a valuable understanding of how brain networks underlying complex linguistic and metalinguistic tasks might develop and change. However, it remains unclear how children's brains respond to systematically increasing challenges to the language production system—for instance, when changes are made in basic psycholinguistic factors such as word frequency, word length, and concept familiarity that are known to modulate both behavior and fMRI activation in adults (Graves et al. 2007; Wilson et al. 2009).

By manipulating multiple levels of complexity in the same task, we can ask how generalizable developmental differences are across the psychological construct (picture naming) and how relatively subtle differences in underlying components of that construct (such as word frequency/length/conceptual familiarity/visual complexity) might be driving the observed developmental differences (or lack thereof). In addition, by combining several experimental conditions with multiple baseline conditions (for instance, both a “resting” baseline and a putatively simple control task matched for basic motor, auditory, and visual demands), we can interpret potential differences in children’s and adults’ activation in a variety of ways that do not rely on the assumption that children and adults are showing identical activation in a control task (for a discussion of this issue, see Brown et al. 2006; Kherif et al. 2009). We can also use such multiple reference points to inform interpretation of regions that show relative deactivation and are often masked out in developmental fMRI studies (this may obscure notable developmental differences, as we shall see below).

The use of multiple levels of difficulty within the same basic task paradigm also allows us to probe how localization and lateralization of function might change with task demands. As noted above, in adults, effects of word frequency and length tend to be observed predominantly (but by no means exclusively) in left frontal, occipito-temporal, and inferior parietal regions (Graves et al. 2007; Wilson et al. 2009). While children show similar behavioral effects of word length and frequency and conceptual familiarity in picture-naming tasks as adults (e.g., longer reaction times and lower naming agreement—D'Amico, Devescovi, and Bates 2001), it is not known whether neural responses to these challenges are similar to those observed for adults.

Here, we explore how school-age children’s brains confront the different challenges of this fundamental linguistic task, how they differ from adults, and how their brain organization changes within the school years themselves. To our knowledge, this is the first larger-scale study of children using an overt language production task that requires no literacy skills or metalinguistic demands, and that incorporates multiple levels of difficulty and baseline conditions. We scanned a fairly large and behaviorally well-characterized cohort of school-age children (*N*= 37) and young adults (*N*= 19) on a straightforward, extensively normed picture-naming task, with 3 levels of difficulty.

We find that while school-age children and young adults do show many commonalities in activation (and condition-specific modulations of activation), there are large and regionally specific developmental differences in activation for picture naming that would not be picked up using one task alone, or one baseline alone. Not only do children and adults show quite different patterns of auditory-related activation potentially associated with speech monitoring (Dhanjal et al. 2008; Simmonds et al. 2011; Agnew et al. 2013; Parker Jones et al. 2013), and differences in higher-order visual areas (Cohen Kadosh et al. 2013), we find that they show fundamentally different responses in frontal regions to incremental increases in naming difficulty.

## Materials and Methods

This study was approved by the UCL Research Ethics Committee.

### Subjects

Fifty-six healthy right-handed children (aged 7.04–12.5 years, 31 male), and 19 healthy adults (aged 19.9–45.4 years, 10 males), gave written informed consent to participate in this study. Forty-four of these children (7.08–12.5 years) were recruited and scanned specifically for this study, and 12 of them (ages 7–7.6 years) were scanned as part of a larger longitudinal study. Subjects were all native English speakers, with normal or corrected-to-normal vision, and with no history of neurological, speech/language or hearing impairment. Following data control quality checks (see Data quality control below), 19 children (13 males) were excluded. The final subject selection was therefore 19 young adults (mean age = 26.37, range = 19.9–45.4, 10 males) and 37 children (mean age = 9.73, range = 7.04–12.5, 18 males).

### Stimuli

One-hundred and forty-four black and white line drawings (depicted against a gray background) were used to elicit naming. These line drawings were selected from the corpus developed at the Centre for Research in Language, University of California, San Diego (Bates et al. 2000). These grayscale images were digitized, resized to a standard of 300 × 300 pixels and presented against a white background. A subset of these line drawings was then selected to include those that were named consistently, with minimum cultural bias. This subset included 90 objects from a range of categories such as household objects, animals, fruits, and vegetables. Based on results from a separate sample (Bates et al. 2000; Iyer et al. 2001; Szekely et al. 2003), stimuli were classified as “easy” or “hard” as determined by reaction time and name agreement (the extent to which different people produce a particular name for a given picture). Easy and hard words were normed and matched for a range of linguistic and visual variables (see Table 1). In addition, meaningless control stimuli served as stimuli for the “silly” condition. These consisted of a set of meaningless line drawings that were selected to match experimental stimuli in complexity, size and luminance, and were not consistently namable. Forty-five line drawings were selected for each run.

**Table 1:** Details of stimuli used from the International Picture-Naming Project (Szekely et al. 2003)

Stimuli	RT target mean	Familiarity	Syllables	Characters	Log frequency CELEX	AOA	Visual complexity
**EASY**							
Apple	810	1	2	5	3.434	1	8241
Balloon	702	1	2	7	1.946	1	8015
Banana	808	1	3	6	2.197	1	8767
Bell	703	1	1	4	3.332	3	11 109
Book	656	1	1	4	6.075	1	8619
Broom	821	1	1	5	2.197	1	11 261
Camera	725	1	3	6	3.611	2	16 408
Carrot	806	1	2	6	2.197	1	13 201
Cat	766	0.96	1	3	4.22	1	9894
Clown	804	1	1	5	1.609	2	21 244
Dog	702	1	1	3	4.754	1	12 012
Eye	700	0.98	1	3	6.261	1	9104
Finger	775	0.98	2	6	4.82	1	5370
Fire	854	0.98	2	4	5.094	3	52 543
Frog	751	1	1	4	2.303	1	14 773
Helicopter	793	1	4	10	2.833	2	18 241
House	745	0.98	1	5	6.409	1	18 069
Kangaroo	856	1	3	8	1.386	3	14 555
Knife	816	1	1	5	3.807	2	8773
Leaf	848	1	1	4	4.407	3	26 600
Moon	804	1	1	4	4.094	1	3730
Pencil	702	1	2	6	2.996	2	7899
Ring	785	1	1	4	1.386	3	7652
Sink	984	0.96	1	4	2.773	1	26 560
Sock	712	1	1	4	2.944	1	8316
Spoon	777	1	1	5	2.773	1	7344
Sun	762	1	1	3	5.03	1	18 102
Table	852	0.98	2	5	5.464	1	12 010
Turtle	734	1	2	6	1.609	1	14 768
tree	796	1	1	4	5.257	1	26 074
**HARD**							
Airplane	778	0.7	2	8	1.946	1	16 810
Alligator	881	0.9	4	9	1.099	2	14 874
Canoe	1164	0.62	2	5	1.946	3	27 029
Dresser	1163	0.48	2	7	1.792	3	21 173
Glove	848	1	1	5	2.996	3	11 509
Gorilla	944	0.7	3	7	1.386	3	17 084
Grasshopper	1234	0.67	3	11	1.386	3	13 119
Ironing Board	1105	0.9	4	12	0	3	12 848
Sweater	1122	0.55	2	7	2.773	1	11 622
Lawnmower	1166	0.96	3	9	0	2	18 238
Leopard	1194	0.54	2	7	2.197	3	23 203
Moose	1158	0.76	1	5	0.693	2	23 330
Nest	1059	0.73	1	4	2.89	3	12 296
Palm tree	908	0.86	2	8	0	3	18 577
Panda bear	1071	0.38	2	5	0.693	3	29 117
Paper clip	1262	0.81	3	9	0	3	21 555
Parrot	910	0.79	2	6	1.609	3	18 115
Piggy bank	965	0.94	3	9	0	3	24 489
Rhinoceros	998	0.77	4	10	1.099	3	18 320
Saxophone	1061	0.81	3	9	0.693	3	8795
Stairs	1011	0.74	1	6	3.807	1	27 602
Stethoscope	1209	0.93	3	11	0.693	3	13 841
Swan	1049	0.74	1	4	2.079	3	12 465
Teapot	1085	0.44	2	6	1.609	3	17 625
Television	786	0.61	2	2	0	1	18 950
Tractor	1216	0.87	2	7	2.485	2	9518
Trumpet	1053	0.69	2	7	2.197	3	13 615
Vase	1171	0.94	1	4	2.079	3	20 221
Violin	1051	0.82	3	6	1.946	3	8571
Spiderweb	869	0.68	3	9	0	3	14 705

### Experimental Design

**Figure 1.**
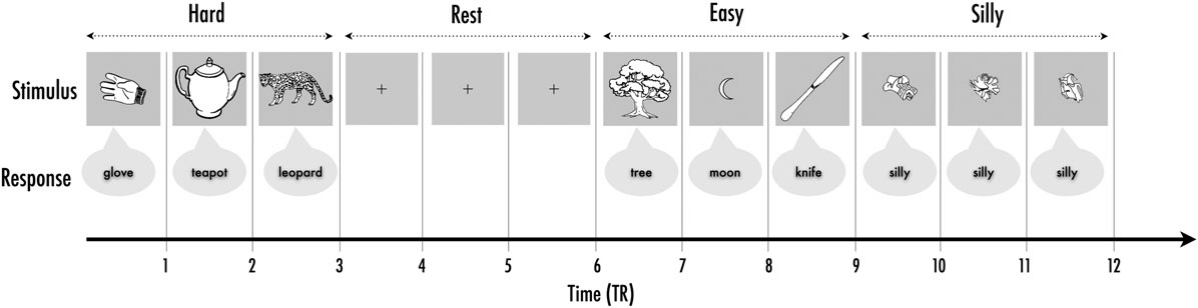
Schematic of experimental design over TRs.

In 2 separate runs, participants were asked to name pictures. Stimuli were grouped into an event-related design, with 3 trials forming a mini-block (to maximize signal-to-noise; Liu 2004). Two baseline conditions were included: the first was a simple fixation baseline, where subjects remained silent and fixated on a cross (henceforth “rest”), and the second presented different meaningless line drawings, in response to which subjects were instructed to say “silly” (nonsense picture labeling, henceforth “silly”). In each run, there were 5 mini-blocks of these 4 conditions (hard, easy, silly, and rest trials). A run was 4.58 min long. There were 60 trials per run that included 15 trials from each condition (hard, easy, silly, and rest). Easy and hard-to-name pictures were grouped into mini-blocks, alternated and interspersed with the 2 baseline conditions (“rest” and “silly”). The order of runs was counterbalanced across subjects. Figure 1 shows a schematic of the experimental design.

### MRI Acquisition

MR data were collected with a 1.5T Siemens Avanto scanner with a 12-element receive-only head coil. Functional data consisted of 27 *T*_2_*-weighted echo-planar image slices (repetition time [TR] 4560 ms, echo time [TE] 41 ms, field of view 224×224 mm), giving a 3.5×3.5×3.5 mm resolution. A semi-sparse sampling design was used to limit the effects of jaw and tongue motion on the B0 field. The length of the silent period (after the presentation of the picture) was 1500 ms. Prospective motion correction (Siemens PACE) was also applied during functional scanning. Oblique axial slices were automatically positioned using Siemens AutoAlign. The AutoAlign protocol acquires several short anatomical images at the beginning of each scanning session to align the participant's brain to a standard template brain, where the slice planes are defined. The slices were aligned to focus on temporal and inferior frontal areas, and did not fully cover the superior frontal and the posterior occipital/ cerebellar regions (see Supplementary Fig. 1). A total of 64 volumes were collected per run. An automated shimming algorithm was used to reduce magnetic field inhomogeneities. In addition, for anatomical localization purposes, a *T*_1_-weighted MPRAGE scan (magnetization prepared low angle spoiled gradient echo, TE 3.57, TR 2730 ms, field of view 224 × 256 mm) was acquired during the scanning session with 1 mm in-plane resolution and 1 mm slice thickness (176 slices collected). For the 12 children scanned as a part of a larger longitudinal study, we collected structural scans using a 32-channel head coil.

### Procedure

Participants undertook a short training session with a small subset of the words outside of the scanner to ensure that they understood the task. Within the scanner, they were reminded to name all the pictures overtly with minimal mouth movement. Pictures appeared centered on the projection screen and participants named the picture. If they could not identify the picture, they were asked not to say anything. They were also reminded about the presence of the “silly” pictures, and that they had to say “silly” when then these pictures appeared. Finally, participants were instructed to look at the fixation cross when pictures did not appear and were instructed not to name the cross. Participants completed 2 runs of picture naming, with 15 trials each of the silly, easy, and hard conditions interspersed with rest in each run.

Participant in-scanner responses were audio recorded and coded offline for accuracy. Responses were scored as correct if the response provided was the target or an item from a superordinate category (for instance, “insect” instead of grasshopper). A response was considered incorrect if there was no response, an unrelated response, and if a related but incorrect semantic item was provided (e.g., “flute” instead of “trumpet”).

As part of this study, participants also did 4 other functional runs involving 2 unrelated tasks. These results are not reported in the current paper. These 6 functional runs were presented in counterbalanced order across participants. Participants also completed a series of behavioral tests that assessed auditory-motor and linguistic ability (for further details, see Krishnan et al. 2013).

### Analyses

#### Preprocessing

All functional data were converted to NIFTI format and analyzed using the FMRIB software library (FSL, http://www.fmrib.ox.ac.uk/fsl). The images were flipped into the standard FSL orientation and the nonbrain structures in the EPI volume and the *T*_1_-weighted MPRAGE were removed with FMRIB's Brain Extraction Tool (Smith 2002). After removing the first 4 images of each session to allow for *T*_1_ equilibrium, functional images were motion corrected using MCFLIRT to the middle volume to correct for small head movements (Jenkinson et al. 2002). These images were then spatially smoothed with a 6 mm full-width half maximum Gaussian filter to increase signal-to-noise. Time series data were prewhitened to remove temporal auto-correlation, carried out using FILM with local autocorrelation correction (Woolrich et al. 2001).

#### Data Quality Control

Estimated mean displacement was assessed using MCFLIRT. Only runs with <0.1 mm maximal absolute movements and <0.08 mm relative movement were included for further analysis. Based on these criteria 19 children were excluded from analyses, and a single run was excluded for 13 children. Additionally, to exclude effects of excessive head motion that are not removed by motion correction, the mean absolute deviation from the median was calculated for each volume using AFNI (3DToutcount, http://afni.nimh.nih.gov/afni/). For each run, 6 of the most extreme volumes (10%) were identified. These volumes were modeled in the design matrix as effects of no interest (see Dekker et al. 2011).

#### First Level Analyses

The presence of pictorial stimuli in the present study was modeled (“hard”, “easy”, “silly”) by convolving trial onsets with a double-gamma canonical hemodynamic response function (Glover 1999). Silent trials formed the fixation baseline or “rest” condition. In-scanner responses to the pictures were recorded and were hand-coded for accuracy as described above. Incorrect responses were identified and added to the design matrix as effects of no interest. (There were insufficient incorrect responses to analyze the blood oxygen level–dependent [BOLD] response to these trials). In addition, temporal derivatives and estimated motion parameters were included as covariates of no interest (Jenkinson et al. 2002).

First level results were then transformed into standard space using a 12 degree-of-freedom affine registration by first registering each functional run to each subject's high-resolution structural scan, and then registering the high-resolution *T*_1_-weighted volume to the Montreal Neurological Institute MR atlas average of 152 normal subjects (MNI-152) using FMRIB’s Linear Image Registration Tool (FLIRT). Child brain normalization is an accepted method in many developmental fMRI studies (Brown et al. 2005; Dekker et al. 2011); from 6–7 years of age, standard normalization procedures do not lead to registration problems (Muzik et al. 2000; Kang et al. 2003; Wenger et al. 2004).

Easy, hard, and silly naming trials were modeled in the design matrix with respect to rest. Each functional run for a given participant was modeled separately at the first level. Statistics for the contrasts of interest, averaging across the 2 runs for each participant, were estimated using fixed effects models.

#### Group Level Analyses

At the group level, random-effects components of mixed effects variance were modeled and estimated for each contrast of interest, using FLAME (FMRIB’s Local Analysis of Mixed Effects) Stage 1 with automatic outlier detection (Beckmann et al. 2003; Woolrich et al. 2004; 2008). Voxelwise estimates of the probability of gray matter membership intensity were included as a covariate to control for potential gray matter differences between groups. To identify significant clusters of activation, all *Z*-statistic (Gaussianised *T*/*F*) images were first thresholded using clusters determined by *Z*> 3.1 and a (corrected) cluster significance threshold of *P*= 0.05 (Worsley 2001). The only exception made was for the conjunction analyses across adults and children, where clusters were determined by *Z*> 2.3 and a (corrected) cluster significance threshold of *P*= 0.05 (Nichols et al. 2005). We chose this more lenient—yet still whole-brain-corrected—significance threshold in order to demonstrate shared patterns of activation that may not have emerged within individual groups because of slightly sub-threshold activation within one group or the other.

Six sets of group-level analyses were planned to explore the changes in activation over development for the picture-naming task. We compared activation over groups for main effects (hard, easy and silly relative to rest) and also compared the main effects (easy > silly, hard > silly, and hard > easy). We directly compared the cohorts’ responses on each of these 6 contrasts. For the child cohort, we conducted whole-brain regression analyses with these 6 contrasts using chronological age as a predictor. To simplify the interpretation of our results, we first present the commonalities in activation across the hard, easy and silly conditions, then report commonalities in activation when naming hard and easy pictures relative to silly shapes, and finally present the differences in activation for hard-to-name pictures relative to easy-to-name pictures. We then examine how adults’ and children's neural activation is modulated by increments in naming difficulty.

## Results

### Behavioral Results

As would be expected given previous norming studies (D'Amico et al. 2001; Iyer et al. 2001; Bates et al. 2003; Szekelky et al. 2003), both children and adults were very accurate in naming pictures, but with adults more accurate when collapsed over condition (*F*_(1,54)_ = 10.951, *P* = 0.002). As can be seen in Table 2, this age group effect interacted with condition (*F*_2.067,111.612_ = 5.736, *P* = 0.004). The adult > child accuracy difference was largest in the hard naming condition.

**Table 2:** % Accuracy over rest trials and the 3 naming conditions

Condition	Adults	Children
Rest	100% (0%)	99.6% (1.2%)
Silly	100% (0%)	94.8% (11.5%)
Easy	99.8% (0.8%)	97.6% (4.1%)
Hard	98.8% (2.3%)	88.7% (12.6%)

Note: Standard deviations are indicated in brackets.

Note that only trials that children and adults completed accurately were analyzed in the following fMRI analyses.

### fMRI results

#### Activation Observed for Speech Production > Rest (Fig. 2 and Table 3)

##### Regions showing similar activation in children and adults

When producing words in response to a complex visual figure relative to rest, conjunction analyses (Nichols et al. 2005) showed both groups recruited much of the picture-naming network reported in the existing literature (Price 2012). This included presumptive representations of the supralaryngeal vocal tract in bilateral primary motor and multiple somatosensory areas (Bouchard et al. 2013; Conant, Bouchard, and Chang 2014; Carey et al. forthcoming), anterior superior insula and supplementary motor areas (associated with articulation—Dronkers 1996; Ackermann and Riecker 2004; Baldo et al. 2011), bilateral striate and extrastriate visual areas (including higher-level object processing regions—Dekker et al. 2011; Cohen Kadosh et al. 2013), as well as multiple thalamic and basal ganglia nuclei (Watkins et al. 2002; Riecker et al. 2008). Both groups also showed considerable relative deactivation across conditions in bilateral medial prefrontal cortex, typically thought of as part of the anterior default network (Seghier and Price 2012).

**Table 3:** Peak co-ordinates and *Z*-values for Adult > Child differences from the 3 contrasts (A) Hard and Easy and Silly > Rest, (B) Hard and Easy > Silly, and (C) Hard > Easy, grouped by cerebral lobe and then by contrast

Brain region	*x*, *y*, *z*	Hard and Easy and Silly > Rest	Silly > Rest	Easy > Rest	Hard > Rest	Hard and Easy > Silly	Hard > Easy
Frontal							
L postcentral gyrus/central operculum	**−66**, **−8**,**8**	**5.45**	2.26	0.94	2.97	1.61	2.09
L inferior frontal gyrus, pars opercularis	**−56**, **18**, −**2**	**4.86**	1.57	2.55	2.93	2.82	1.96
R precentral gyrus	**52**, **6**, **44**	**5.02**	3.12	**4.73**	**4.43**	**4.12**	2.27
R inferior frontal gyrus, pars opercularis	**42**, **10**, **22**	3.12	1.52	4.03	**3.38**	**4.58**	0.69
R Frontal Pole	**12**, **60**, −**12**	0.1	−0.66	−1.19	1.93	1.29	**4.18**
Parietal							
R posterior supramarginal gyrus	**62**, **−40**, **22**	**4.38**	**3.67**	3.19	**3.11**	2.43	0.61
L angular gyrus	−**44**, **−60**, **28**	−1.49	−0.62	−3.03	0.04	−1.99	**4.33**
Temporal							
R posterior superior temporal gyrus	**68**, **−18**, **10**	**4.9**	2.05	1.86	2.72	1.46	1.57
R temporal pole	**62**, **12**, −**12**	**4.88**	1.04	2.15	1.52	2	0.99
R middle temporal gyrus, temporo-occipital part	**58**, **−56**, **0**	**4.45**	**4.47**	3.87	**4.09**	2.65	0.52
R posterior superior temporal gyrus	**64**, **−30**, **6**	**3.62**	1.93	**3.77**	**3.65**	**4.6**	1.47
L inferior temporal gyrus, temporo-occipital part	**−44**, **−56**, **−8**	**3.68**	1.71	**3.6**	**3.85**	**4.25**	2.65
L posterior superior temporal gyrus	**−64**, **−36**,**4**	2.15	1.41	0.53	2.72	1.79	**4.34**
Occipital							
L lateral occipital cortex, inferior division	**−58**, **−64**,**−2**	**4.77**	**3.16**	**3.73**	2.36	2.83	−0.8
R lateral occipital cortex (inferior)	**44**, **−80**, **−4**	3.6	3.08	3.61	**4.04**	**4.03**	2.09
Subcortical							
L thalamus	**−18**, **−20**,**12**	**4.21**	3.05	3.97	3.04	2.58	−0.75

Note: *Z*-values that are in bold are significant at a whole-brain level; others are provided to show patterns of results over regions.

**Figure 2.**
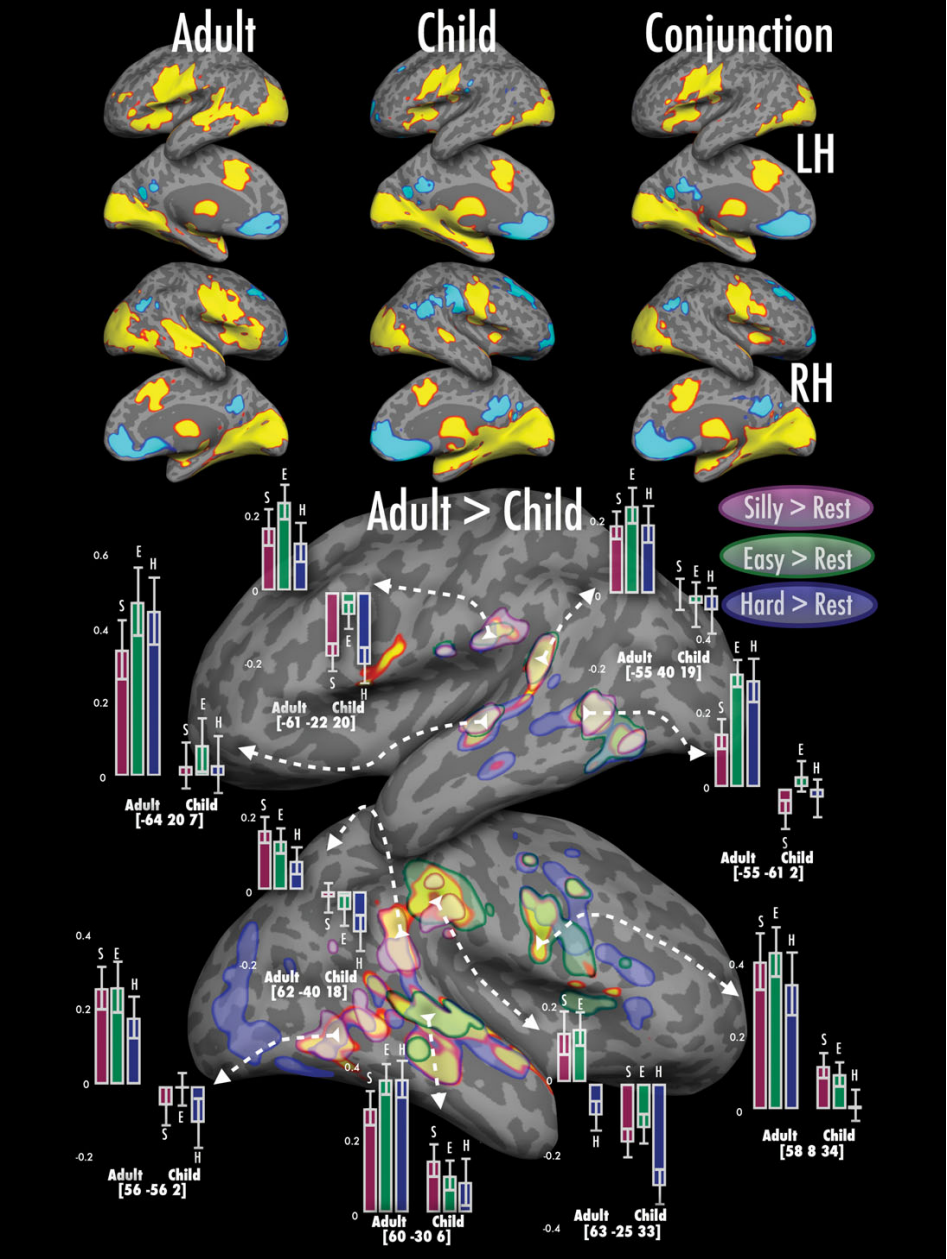
Effects of age group (adults/children) on producing speech in response to a complex visual figure relative to rest. Children and adults show bilateral activation in the inferior portion of the central sulcus (extending over pre- and postcentral gyri), anterior insulae, anterior cingulate cortex and left IFG. As might be expected given the presence of pictorial stimuli, increased activation is also observed in bilateral primary and higher-level visual regions such as medial and lateral occipital regions as well as the lingual gyri. Subcortical activation is observed in multiple thalamic regions, caudate and putaminal nuclei and the ventral diencephalon (not shown). Deactivation relative to rest is observed in the medial prefrontal cortex, precuneus and posterior cingulate cortex bilaterally as well as in right posterior superior temporal sulcus and right superior frontal gyrus. Adults show greater activation relative to children in bilateral superior temporal gyri, precentral gyri and parietal operculum. The outlines denote significant thresholded group differences for naming hard-to name pictures (blue), easy-to-name pictures (green) and silly shapes (maroon) specifically. Note: in all figures, overall thresholded activation maps (*Z* > 3.1, whole-brain cluster-corrected level of *P*< 0.05) for each group are registered to and displayed on an adult's cortical surface in FreeSurfer (normalized to the MNI-152 template). Lateral and medial surfaces for both hemispheres are shown, subcortical activation is not shown. Maps are colored using a heat scale—red/yellow colors represent increases in activation and turquoise/blue represent decreases in activation relative to the baseline (only activation over threshold is shown). Across conditions, the first column illustrates the overall pattern activation in adults, the second column depicts activation in children, and the third column shows results of the conjunction analyses showing commonalities in activation for children and adults at *Z*> 2.3 at a whole-brain cluster-corrected level of *P*< 0.05. The 2 larger images at the bottom illustrate group differences; adults always show greater activation than children. Bar graphs show % signal change in a 4 mm-sphere centered on a voxel picked for illustrative purposes, bars separated by group (adult/child); error bars show ±1 standard error.

##### Regions showing more activation in adults than in children

In all conditions (relative to rest), adults showed more activation than did children over a subset of the “mature” or adult picture-naming network. Adult > child activation differences were observed over both hemispheres, including in higher-order visual regions (posterior middle and inferior temporal gyri), auditory and audiomotor regions (lateral superior temporal gyri) presumptive ventral premotor (PMv) regions (precentral gyri) and orofacial somatosensory regions (supramarginal gyri). Generally, adults showed suprathreshold activation above resting baseline in these regions, whereas children showed no significant activation, or significant deactivation relative to resting baseline.

However, as illustrated by the translucent colored patches and bar graphs in Fig. 2, the extent and magnitude of this adult > child effect was modulated to some degree over conditions, particularly in the right hemisphere. We discuss this in more depth in Quasi-parametric modulation of activation related to naming complexity (Hard > Easy > Silly)—similarities and differences between children and adults (Fig. 6), below.

#### Activation Observed for Naming Familiar Objects > Saying “Silly” (Fig. 3 and Table 3)

##### Regions showing similar activation in children and adults

When viewing either easy—or hard-to-name real objects compared with nonsensical (silly) shapes (thereby controlling to some degree for common lower-level visual and sensorimotor processing), both adults and children showed greater activation in bilateral early visual and left lateral occipital object-related visual areas. Both groups also showed activation in orofacial somatosensory and motor regions, including presumptive PMv areas, the bilateral insulae and left IFG that are typically associated with complex articulatory processing and word retrieval (Price 2012) as well as increased naming latencies (Graves et al. 2007). Patterns of activation specific to easy > silly and hard > silly are depicted in Supplementary Fig. 2.

**Figure 3.**
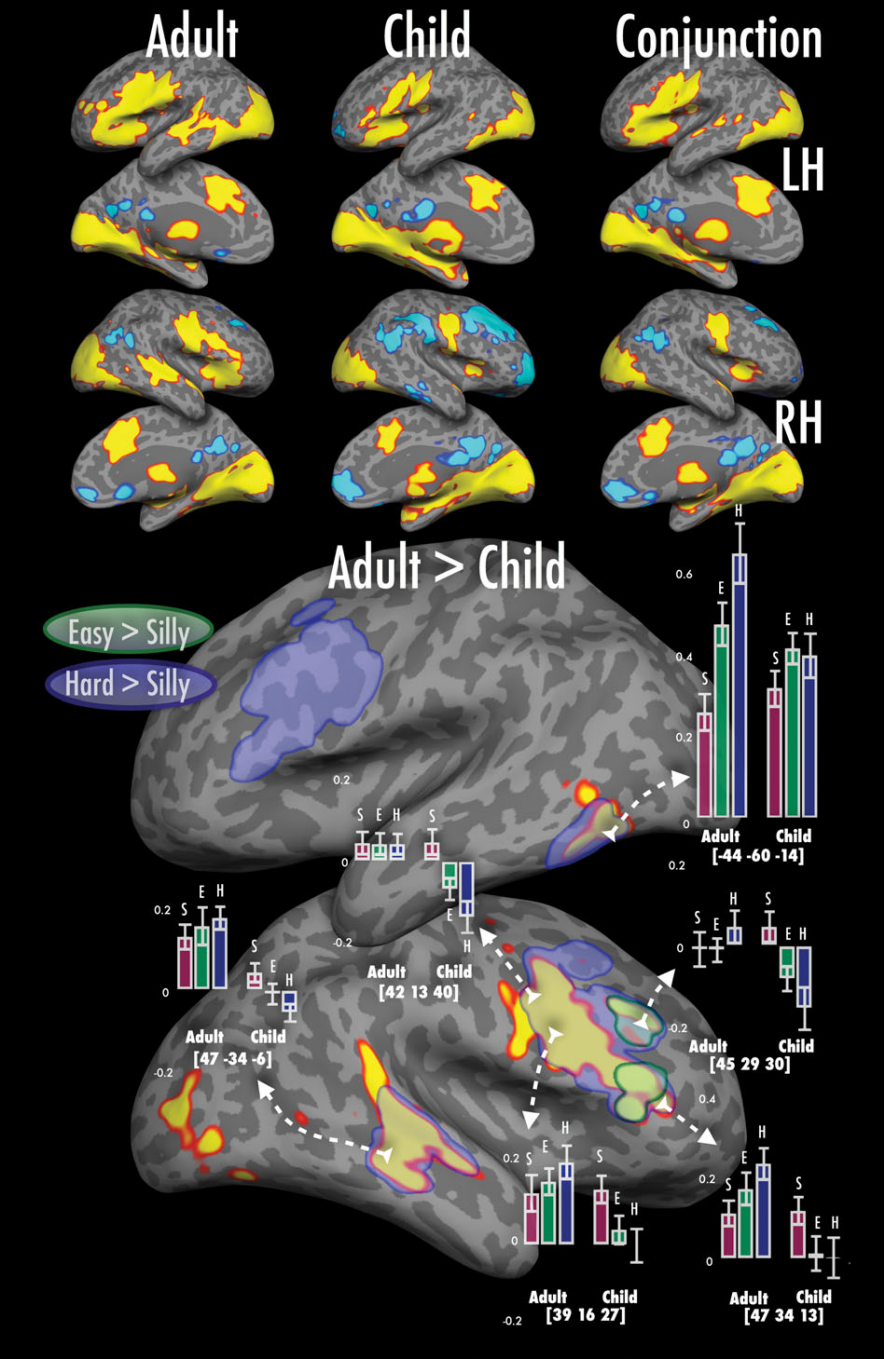
Effects of age group on activation for naming familiar objects relative to unfamiliar nonsensical shapes. Adults and children show common activation along bilateral medial and lateral occipital cortex, anterior cingulate cortex, anterior insulae, the inferior part of the precentral gyrus as well as the left IFG and superior temporal gyrus. Subcortical activation is observed over in multiple thalamic regions as well as caudate and putaminal nuclei bilaterally (not shown). Relative to “silly” there is less activation for easy- and hard-to-name pictures in the right superior frontal gyrus, posterior-most superior temporal sulcus and medial prefrontal cortex as well as bilateral precuneus. Adults show a greater positive difference in activation in the right inferior/middle frontal gyri, the middle part of superior temporal sulcus and left inferior temporal gyrus. The outlines denote significant thresholded adult > child group differences for naming hard-to-name pictures relative to silly shapes (blue) and easy-to-name pictures relative to silly shapes (green). The outline over left inferior and middle frontal gyri is further illustrated in Figure 6.

##### Regions showing more activation in adults than in children

There were several regions where adults showed a greater positive difference in activation compared with children for naming objects versus “silly”. In the left posterior inferior temporal gyrus, adults showed considerably more activity for naming objects compared with silly, whereas children showed a smaller difference between these conditions. Along the right IFG and right prefrontal cortex, adults showed more activity for naming versus silly, whereas children showed the opposite pattern (silly > naming); this pattern of activation was also observed in the right mid superior temporal sulcus (STS), a region often associated with speech processing. Finally, at the posterior aspect of the middle frontal gyrus, adults showed no hint of a difference in activation between naming and silly, whereas children again showed a silly > naming profile of activation (see Quasi-parametric modulation of activation related to naming complexity (Hard > Easy > Silly)—similarities and differences between children and adults (Fig. 6) for detailed naming complexity-related analyses).

##### Regions showing changes in activation within the school years (child cohort only)—Figure 4 and Table 4

While we found no whole-brain-corrected significant relationships between school-age children's chronological age and activation for simple contrasts (i.e., versus rest), chronological age *did* predict activation for the easy > silly and hard > silly contrasts in several regions. (These correlations were just slightly below cluster-corrected threshold in the combined contrast of naming hard and easy pictures relative to “silly”, except in the left precentral gyrus). Here, with increasing age, there was an increase in activation in both contrasts along the opercular part of the left IFG, left middle frontal gyrus, and middle anterior cingulate gyrus extending onto the medial superior frontal gyrus. In the easy > silly contrast, this positive correlation with age extended onto the inferior part of the left precentral gyrus laterally and more superiorly and inferiorly on the medial surface.

**Table 4:** Peak cluster co-ordinates corresponding to age-related differences in children

Effect	Brain regions	MNI (*x*, *y*, *z*)	Cluster size	*Z*
(A) Hard > silly	L superior frontal gyrus	−2, 32, 44	231	4.03
	L inferior frontal gyrus	−44, 24, 20	182	4.02
(B) Easy > Silly	L inferior frontal gyrus	−52, 28, 22	347	4.60
	L paracingulate gyrus	−4, 38, 30	307	4.55
	L frontal pole	−12, 56, 40	296	4.46
	L precentral gyrus	−44, 2, 30	185	4.04

**Figure 4.**
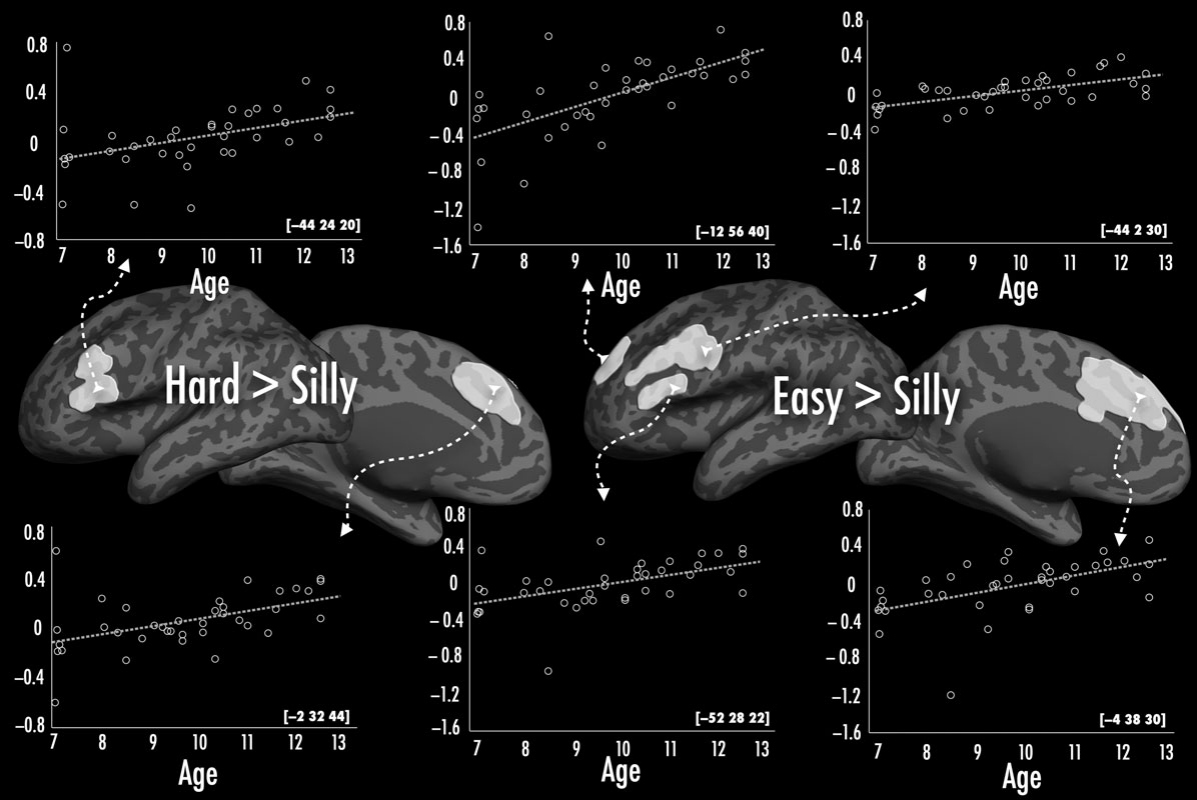
Positive correlations between age and activation in hard > silly over the opercular part of the left IFG and the left superior frontal gyrus and easy > silly over the left frontal pole, IFG, precentral gyrus and paracingulate gyrus are illustrated using scatterplots (shown in white). The *Y*-axis of scatterplots represents % signal change, and the *X*-axis represents age in years. Arrows indicate location of peak voxel in the cluster.

These age-related correlations within the school-age children converged to some degree with the adult–child differences we observed, in that adults had a greater positive difference in activation compared with children over the left IFG and middle frontal gyrus in hard > silly contrast (but not in the easy > silly contrast).

#### Activation Observed for Naming Hard > Easy Pictures (Fig. 5 and Table 3)

##### Regions showing similar activation in children and adults

As noted above and depicted in Table 1, hard-to-name pictures differed relatively subtly across several dimensions from easy-to-name ones, including the frequency of word occurrence, phonological complexity, and visual object complexity. In both adults and children, harder-to-name pictures evoked additional, strongly left-lateralized activation across the IFG and anterior insula compared with easy-to-name pictures (as well as nonsensical pictures). Such increases in the left IFG and anterior insula have been associated with naming lower frequency words (Graves et al. 2007; Grande et al. 2011) and with increased naming latencies (Wilson et al. 2009). The hardest-to-name pictures also drove greater relative *deactivation* in multiple posterior “default” areas (Leech et al. 2011; Leech et al. 2012), as well as in somatosensory areas in the anterior supramarginal gyrus that are typically associated with mouth and face sensation (Huang and Sereno 2007). We discuss a potential mechanism behind this difficulty-related somatosensory deactivation below.

**Figure 5.**
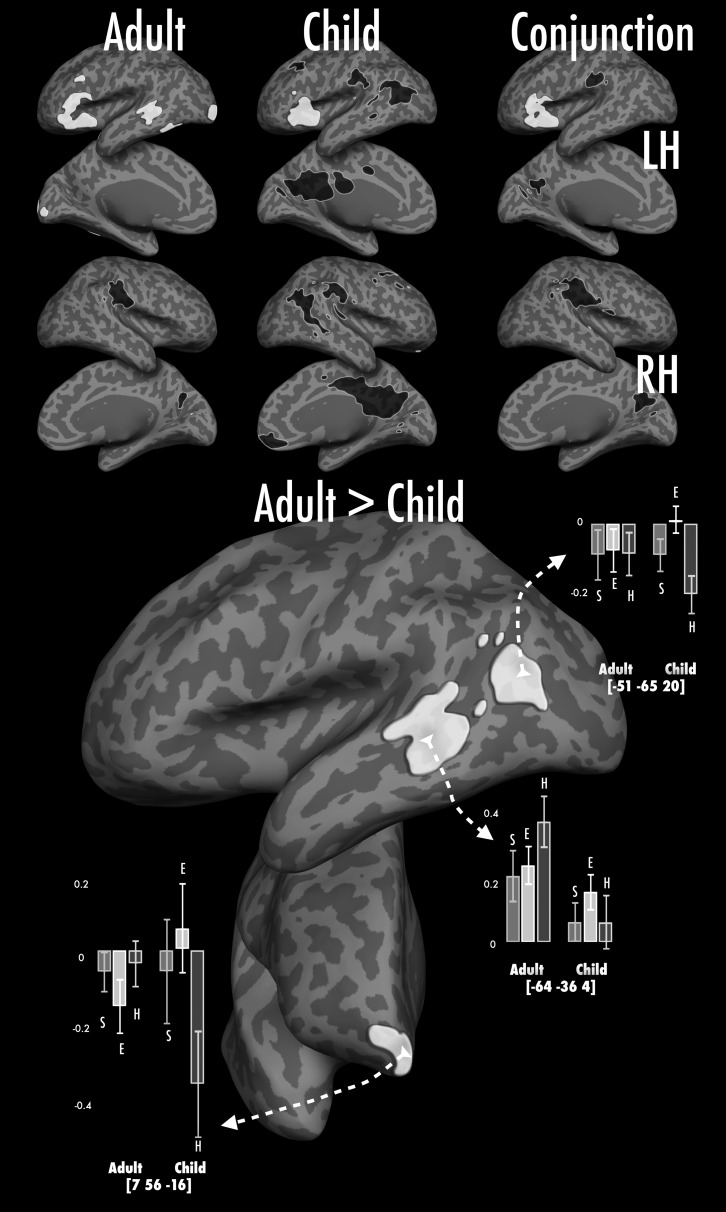
Effects of age group on activation for naming hard pictures relative to easy pictures. In this graph alone, activation is depicted in white and relative deactivation in black. Relative to easy-to-name pictures, hard-to-name pictures were associated with increased activation over the IFG in both adults and children. Less activation for hard-to-name pictures (compared with easy-to-name pictures) was observed in the supramarginal gyri and precuneus bilaterally. Adults show a greater positive difference in activation in left middle temporal gyrus/angular gyrus, the posterior part of the left superior temporal sulcus and the right frontal pole.

##### Regions showing more activation in adults than in children

Adults showed a significant greater positive difference in activation than children in 3 regions, all of which had differing profiles of activation. In the posterior-most aspect of the left middle temporal gyrus (often implicated as part of the default mode network), adults showed no sign of a hard versus easy difference (with both under baseline levels), whereas children showed no significant activation for easy naming, and deactivation compared with baseline for hard naming. In the left STS inferior to the planum temporale, in a region often involved in speech comprehension (Leech et al. 2009), adults showed more positive-going activation for hard to name pictures compared with easy (with both well above baseline), whereas children showed more activation in the easy compared with hard naming condition. Finally, along the right frontal pole, adults showed more deactivation for easy than hard naming (relative to baseline), whereas children showed the opposite pattern. (It is worth noting that there was a great deal of individual variability in activation in this region, so we approach this result with some caution).

#### Quasi-Parametric Modulation of Activation Related to Naming Complexity (Hard > Easy > Silly)—Similarities and Differences Between Children and Adults (Fig. 6)

As noted above, the “silly”, “easy”, and “hard” naming conditions represent a stepwise modulation of difficulty or complexity, where demands on lexical retrieval, phonological production, and visual object processing increment over each condition. Here, we first asked whether there were brain regions in *both* children and adults that showed monotonic (increasing *or* decreasing) activation as a function of naming complexity. In a second analysis, we asked whether some brain regions showed different responses to naming complexity across children and adults, for example, regions where children would show monotonic complexity-related *decreases* in activation, but adults would show complexity-related *increases* in activation.

**Figure 6.**
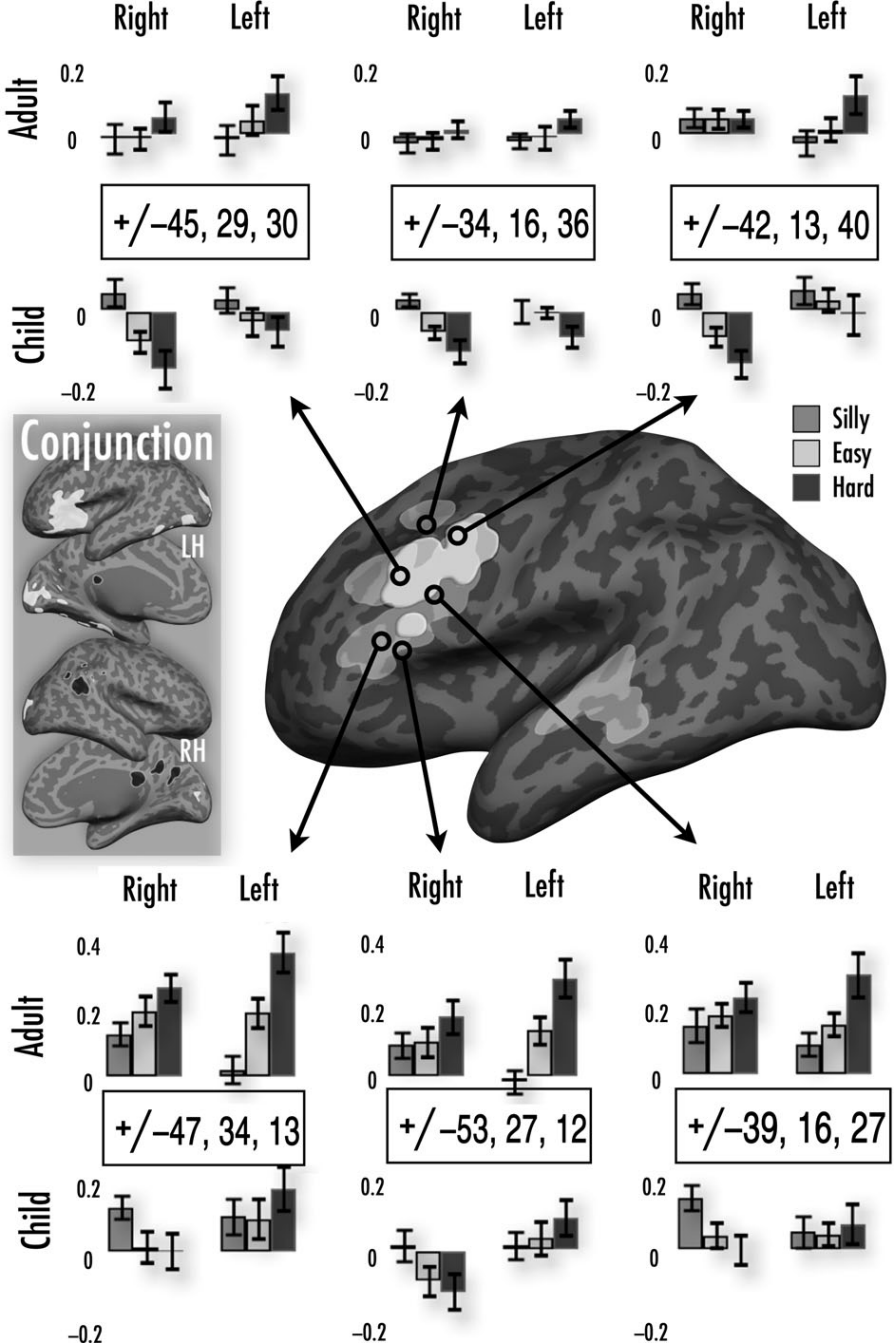
Figure depicting responses to complexity across adults and children. The lighter gray overlay represents group differences in hard > silly that show the pattern hard > easy > silly in adults, and hard < easy < silly in children in the right hemisphere (see Results, part D). The brighter white overlay represents the same for the left hemisphere. Voxels are picked to illustrate different patterns of responses across both hemispheres in adults and children. As before, bar graphs show % signal change in a 4 mm-sphere centered on the voxel, bars separated by hemisphere and group; error bars show ±1 standard error. The conjunction inset shows the common increments across adults and children with increasing naming complexity in white and decrements in black.

For the first analyses, we created 2 separate sets of statistical masks for children and adults, one showing positive increments in activation with naming complexity, and the other negative increments in complexity. Regions showing positive complexity-related activation were identified by inclusively masking the adult/child hard > silly conjunction map (from Activation observed for naming familiar objects > saying “silly” [Fig. 3 and Table 3] above) to include only voxels showing positive activation for the easy > silly and hard > easy contrasts in both children and adult groups. (We did not impose any magnitude thresholds on the inclusive masks as we had no *a priori* expectation that the easy-silly difference in processing demands would be the same as the hard-easy difference). Regions showing negative complexity-related activation were those showing the opposite profile (child/adult conjunction of silly > hard, inclusively masked by voxels where activation showed silly > easy and easy > hard patterns in each group).

As shown in the inset of Fig. 6, both children and adults showed increments in activation with increasing naming complexity along the entirety of the left IFG continuing into the anterior insula. Multiple regions related to visual and object processing also showed complexity-related activation, including patches in left posterior and anterior ventral temporal cortex, bilateral lateral occipital regions, and presumptive early visual areas medially in the left hemisphere.

In the right hemisphere, there were multiple regions where both children and adults showed progressive *decrements* in activation with increasing naming complexity, including patches in the supramarginal and angular gyrus, and medially in the precuneus and retrosplenial cortex (with the latter showing a homologous activation in the left hemisphere). All of these tend to be associated with default mode network activity and/or changes in attentional state and cognitive control (Leech et al. 2011).

For the second analysis (showing age-group-related differences in complexity-related activation), we first identified regions that showed a significant effect of age group in the hard > silly contrast, then masked this to include only voxels where adults showed positive activation and children showed negative activation for the hard > easy and easy > silly contrasts (and vice versa).

Figure 6 depicts the opposing responses of adults and children when confronted by increasing task difficulty. Adults had bilateral (but left-lateralized) increases in activation not only in the IFG (as noted above, and as observed as well in children), but in presumptive PMv areas within the inferior frontal sulcus (IFS). Adults also showed more strongly left-lateralized complexity-related activation increases in dorsolateral prefrontal (DLPF) cortex along the middle frontal gyrus, in regions often associated with word retrieval processes (Price 2010). In the same regions, children showed a very different response to increases in task complexity. In the left IFS, children showed little to no difference in activation across conditions, while in left DLPF, there was a slight trend for complexity-related decreases in activation. Finally, in the right hemisphere homologs, children showed very considerable complexity-related decreases in activation.

## Discussion

### Overview

While neural organization for picture naming was in many ways conserved from school age to early adulthood—both in overall patterns of activation and in response to levels of naming difficulty, as shown in conjunction analyses—there were also remarkable differences in the way in which children's and adults’ brains responded to increased naming demands. Perhaps most striking were the effects in inferior and prefrontal cortex, where adults typically showed complexity-related *increases* in activation (hard > easy > silly), while children showed complexity-related *decreases* in precisely the same regions (silly > easy > hard). This was in contrast to the bilateral anterior insulae, where both children and adults had strong complexity-related activation increases.

Developmental differences that were less related to complexity also emerged in regions typically associated with self-monitoring during speech production (posterior superior temporal gyrus and parietal operculum) and higher-level visual object processing (lateral posterior temporal and occipital regions). Here, adults showed considerably more activation in all conditions compared with children, who tended if anything to deactivate relative to the resting baseline.

### Children and Adults Have Opposing Responses to Naming Complexity in Left and Right Frontal Regions

When confronted by increasing task difficulty, adults and children showed diametrically opposed differences in the response to complexity in bilateral prefrontal cortex and right superior temporal gyrus. In considering these group differences, it is important to note that children were able to perform the task and only accurate trials were analyzed. Therefore, the neural differences we observe are likely to indicate different strategies employed by children and adults rather than reflecting different performance.

A potential explanation for these complexity-dependent developmental changes is that prefrontal regions change their functional role over development (perhaps related to the structural changes that continue beyond childhood in prefrontal regions, Sowell et al. 2003; Gogtay et al. 2004). For example, in children these regions may behave more similarly to the default-mode network—and perhaps be functionally coupled to these regions. In contrast, in adults the DLPF cortex is more associated with the cognitive-control network (Dosenbach et al. 2008). There is evidence for developmental change in these regions, which are typically associated with multimodal cognitive control in adults. For instance, Marsh et al. (2006) have shown that there is an increase in activation with age over the middle frontal gyrus when doing a Stroop colour-naming task, which points to the changing role of this region in tasks where suppression or inhibition is required.

With respect to the picture-naming task itself, adults are likely to have greater experience than children with the hard-to-name pictures and associated words, and also have denser lexical neighborhoods (Graves 1986; Anglin 1993; also reviewed in Borovsky et al. 2012). Adults might use regions in bilateral prefrontal cortex to suppress other relevant targets and select the appropriate response. In adults, the right VLPFC has been associated with recollection of *perceptual* details of an object (relative to conceptual details), whereas the left VLPFC shows the opposite pattern (Badre and Wagner 2007). Further, right DLPFC activation is thought to reflect the engagement of processes supporting postretrieval monitoring using episodic and semantic memory (Hayama and Rugg 2009). Adults might automatically activate right prefrontal regions due to their more elaborate perceptual and semantic representations.

On the other hand, we found that children show complexity-dependent decreases over the right ventrolateral prefrontal cortex and bilateral DLPF cortex. Children are likely to have fewer lexical competitors to choose from, and therefore may not require the same levels of inhibition of alternative lexical candidates. Indeed, it may be that children require a “release of lexical inhibition” so as to allow the infrequently encountered lexical candidate to surface from memory. We tentatively speculate that such a release from inhibition for “hard” words would drive the decrease in activation associated with increased lexical retrieval demands. Indeed, a lack of cognitive control has been hypothesized to be beneficial during language learning (Novick et al. 2010; Chrysikou et al. 2011, 2013). Our results may suggest that for overt naming, children may not monitor or apply such higher-level control to their productions after word retrieval in the same way as adults.

The imaging literature on semantic memory in adults also draws a distinction between the functional roles of different parts of the *left* ventrolateral prefrontal cortex (l-VLPFC). The anterior part of the l-VLPFC (roughly coextensive with the left IFG-orbitalis) is thought to be involved in controlled lexical retrieval, whereas the left mid-VLPFC (∼IFG-triangularis) is thought to be responsive to postretrieval selection demands, or alternatively demands on cognitive control (Kan and Thompson-Schill 2004; Badre and Wagner 2007; Schnur et al. 2009). In our study, whereas both children and adults show complexity-related increases in activation in the orbital part of left IFG, children do not show as great a complexity-related increase as adults in the triangular part of the left IFG (see Supplementary Table 1). Again, this may indicate a reduced effect of cognitive control or postretrieval selection demands in childhood.

Along with other studies that show increasing activation over the left frontal cortex with age, we found regions over the left inferior, middle, and superior frontal cortex where activation positively correlated with age (see Fig. 4). However, these were only observed when contrasting activation for naming pictures to the higher-level baseline (e.g., “silly”), but not for any of the other contrasts. These results are consistent with Brown et al. (2005) who observed age-related increases in frontal cortex and interpreted them as evidence for an increase in “top-down” control mechanisms with age. These correlations are suggestive of ongoing change in left frontal regions within the school years. Given our group differences in these years are largely over the right hemisphere, it is likely that the right hemisphere homologs of these regions will undergo similar changes later in development. Indeed, Chou et al. (2006) and Booth et al. (2003) report age-related changes over the right IFG in 9–15-year-old children for semantic tasks (and both studies use high-level baselines).

### Opposing Patterns of Activation for Hard-Versus-Easy Naming Complexity in Children and Adults in Left Posterior Temporal Regions

As in the frontal regions discussed above, adults showed more activation for naming hard pictures versus easy ones, while children displayed the opposite pattern. But unlike the frontal regions, identifying the nonobjects as “silly” fell somewhere in between for both groups. The 2 left temporal regions showing this crossover interaction have quite different functional roles, and correspondingly, different profiles of activation for naming.

In the left posterior STS (pSTS), adults show more activation for hard versus silly pictures (with both above baseline), while children show less activation overall, and the converse pattern. This left pSTS region is often associated with speech comprehension and categorization (Desai et al. 2008), auditory nonspeech object category learning (Leech et al. 2009), as well as speech intelligibility independent of the form of acoustical degradation (Davis and Johnsrude 2003). While it is logical that hearing longer and more phonologically complex words (i.e., “hard”) would evoke greater activation in this region in adults, it is frankly unclear why this region would yield a decrease in activation in children. One possibility is that children may be showing decreased activation for words that are less phonologically familiar. Another speculation is that the pSTS is functionally coupled with the frontal regions showing such large-scale developmental shifts in response to complexity, and that “top-down” effects from the frontal to pSTS regions may drive this opposing pattern of activation. We are currently exploring such possibilities using functional connectivity analyses.

Over posterior middle temporal gyrus/angular gyrus, children and adults both show decreases in activation. The difference in activation is similar for adults over all 3 naming conditions; however, in children, relative deactivation is modulated by complexity (greater deactivation for hard > silly > easy). This region is known to intersect with the default-mode network (Price 2012). Our results in this region are reminiscent of Seghier, Fagan and Price's findings (2010) of increased deactivation over the mid-angular gyrus during an overt picture-naming task. Seghier et al. (2010) speculate that this may be related to the demanding nature of the picture-naming task versus the other reading tasks in their study. It is possible that children's activation in this region is particularly influenced by the demands on semantic and conceptual familiarity (hard > silly > easy), unlike adults who have a similar profile of activation for all 3 naming conditions.

When hard-to-name pictures are compared with easy-to-name pictures, we also see group differences over the right frontal pole. However, given the variability in activation in this region in the present study, future replication is needed to confirm whether this difference is genuinely driven by task-related change.

### Age Group Differences in Orofacial Somatosensory Regions

Regardless of condition, adults showed greater activity than children over both the bilateral supramarginal gyri (except for the hard > rest condition in the right, see Supplementary Fig. 1). These regions are typically associated with somatosensory representations of the lip and face (Huang and Sereno 2007; Huang et al. 2012). The magnitude of this adult > child difference was not significantly modulated by whether children were naming silly, easy, or hard pictures. For silly and easy to name pictures, group differences across conditions were driven by activation in adults and deactivation in children. However, in the “hard” naming condition, adults also showed significant decreased activation over the right supramarginal gyrus relative to the easier conditions, and a nonsignificant trend for less activation in the left homolog.

The fact that adults are showing difficulty-related *decreases* in activation in these presumptive mouth somatosensory areas is reminiscent of the finding of Dhanjal et al. (2008) who showed activation *suppression* in this same region for propositional speech relative to jaw and tongue movement. Dhanjal et al. speculate that this deactivation may be due to suppression of orofacial somatosensory feedback with increasing linguistic demands. Therefore, given that naming is more challenging for children (based on behavioral studies), we might expect that even naming simple pictures—and certainly more demanding ones—might provoke a similar attentional suppression of somatosensory feedback.

Our results are potentially consistent with those obtained by Brown et al.'s (2005) word generation tasks, where there was an age-related activity increase in a similar left SMG region. However, Church et al. (2008) found the converse effect for repetition and reading tasks, where children showed “more” activation in left inferior SMG than adults. Crucially, the word generation tasks of Brown et al. as well as the reading/repetition tasks of Church et al. differ in terms of demands in *lexical retrieval*: word generation requires lexical retrieval, but word repetition and reading do not. Drawing an inference from the combination of these findings with the results from the present study and those of Dhanjal et al. (2008), we hypothesize that it is specifically increasing demands on lexical retrieval in children that drives such attentional *suppression* of somatosensory representations.

### Differences in Auditory and Audiomotor Regions

There were considerable age group differences in activation of auditory and audiomotor-related regions along the superior temporal gyrus (STG). Consistent with previous naming studies, adults showed robust bilateral activation along much of the STG, whereas children showed very little if any difference relative to the resting baseline condition. (The one slight exception to this is in easy naming, where children show relatively weak (subthreshold) activation along lateral STG, as revealed by the conjunction analysis). We tentatively suggest that this difference might be due to a greater role in adults for feedforward auditory prediction and online correction. A recent behavioral study (Shiller, Gracco, and Rvachew 2010) suggests that while children are able to compensate for auditory feedback perturbations during speech output, they do not change and update their perceptual representations of the phonemic boundaries in the same way that adults do in response to these changes in speech output.

The adult > child difference in activation along the STG was not observed in either the Brown et al. (2005) study of verb, rhyme, or opposite generation, nor in the Church et al. (2008) study of repetition and reading. This difference might be due to the fact that both studies used an auditory word cue in half of the trials. It may also be explained in part by the use of continuous EPI in both the Brown et al. (2005) and Church et al. (2008) paradigms, versus the sparse sampling in the current experiment, where talkers’ utterances are not masked by acoustic scanner noise.

### Higher-Level Visual Areas

Adults showed greater activation than children in bilateral inferior and middle temporal gyrus as well as right lateral occipital cortex. Our results are consistent with those of Turkeltaub et al. (2008), one of the only other studies that has examined developmental differences in object naming (albeit as a baseline). In their study, analyses were confined to the ventral occipito-temporal cortex. However, across this region, they observe greater activation for adults in both hemispheres for both letter and object naming. Our results are also consistent with those of Cohen-Kadosh et al. (2013) who find age-dependent activation over very similar regions in the right inferior temporal gyrus in a face emotional expression task. Taken together, these results indicate that these developmental differences related to age may not be confined to a single task, but observed across tasks that involve individuating objects (notably, Dekker et al. 2011 found no age-related differences in these regions when children and adults passively viewed complex objects).

### The Use of Multiple Baselines to Characterize Developmental Change

The baseline-contingent differences in age and age-group effects point to the value of using multiple baseline conditions in developmental studies. In our study, complexity-dependent differences would have been missed had we used only easy-to-name or hard-to-name pictures, or not included different baselines. Perhaps more importantly, we would not have noted some of the most dramatic developmental changes if we had looked only at task-positive activation, as is often reported in developmental studies. Our results—as well as those from a developmental fMRI study on lexical decision (Moore-Parks et al. 2010), suggest that task-induced relative deactivation is quite prominent in children. Indeed, relative deactivation is known to change with age in default areas within language tasks (Sun et al. 2013).

Given that other studies have shown differences in resting-state functional connectivity across development (Fair et al. 2007; Supekar et al. 2009) it is not clear that a resting baseline is fully comparable across children and adults. Alternative active baseline conditions have also been used in developmental fMRI studies (Szaflarski, Holland et al. 2006; Szaflarski, Schmithorst et al. 2006; Raizada et al. 2008; Lidzba et al. 2011). However, it is important to ascertain how activation to the higher-level baseline condition itself might change over age. In the present study, adults and children did not differ significantly in activation for the “silly” baseline in the prefrontal regions where we observed the age-related complexity differences (see Supplementary Fig. 1).

The use of multiple baselines can potentially allow for more comprehensive exploration of developmental differences in task-related activation. As noted previously, there is particular debate about whether activity over Broca's region increases with age. Therefore, we investigated how different baseline conditions might have an impact on activation changes in the 3 task conditions in the opercular, triangular, and orbital aspects of the left IFG. Here, a comparison of each naming condition to resting baseline did not show any significant differences between adults’ and children's activation in any subregion of the left IFG. However, when comparing hard-to-name pictures to “silly” pictures, we observed greater activation for adults relative to children across the triangular part of the IFG (with all the tests above False-Discovery-Rate-corrected for 18 comparisons—Benjamini and Hochberg 1995; see Supplementary Table 1). Thus, the answer to the question of “Are there developmental changes in Broca's region activation?” is contingent upon the comparison condition used.

Indeed, the age-related changes in Broca's region activation over 7–12 years appear to be contingent upon the choice of baseline. As noted in Results, we saw age-related increases in activation in left inferior and prefrontal regions for the hard-silly and easy-silly contrasts (but not for naming relative to rest). When we restrict our regions of interest to the left IFG, we do see the age-related increase in activation for both easy-silly and hard-silly in the orbital part of IFG. However, this effect appears to be driven primarily by significant age-related *decreases* in activation for naming silly pictures versus rest in both orbital and triangular IFG (False-Discovery-Rate-corrected for 18 comparisons; see Supplementary Table 2).

### Skill-Learning Versus Interactive Specialization

Two prevailing views of developmental neural change are “skill-learning” and “interactive specialization” (Johnson 2011a). From a skill learning or alternatively expertise view of development, cortical functional differentiation and specialization reflect in large part the complexity and task demands that particular “special” skills like face and speech perception place on the learner—and above all, the amount and intensity of experience required to acquire that skill. Thus, one prediction of this perspective is that children—who necessarily have less experience and expertise in retrieving and articulating words—might show differences in cortical organization for language production similar to those observed in adults who are speaking a second or later-acquired language.

In a study analogous to the present one, Parker Jones et al. (2012, 2013) compared monolingual English speakers and bilingual nonnative English speakers on a series of fMRI tasks requiring overt speech production—picture naming, reading, saying 1–2–3 to unfamiliar nonobjects and saying 1–2–3 to meaningless letter strings (in triads). Under a skill-learning account, we might expect that activation comparisons of nonnative to native speakers should pattern in the same way as the present study's comparison of children versus adults. In particular, nonnative speakers should show less activation in bilateral DLPF regions compared with native speakers. However, this prediction was not borne out. Instead, adults naming pictures in their second language did not differ from native speakers in their activation in these regions (Parker Jones, personal communication). In fact, the bilinguals showed greater activation than monolinguals over the inferior frontal cortex (Parker Jones et al. 2012). These results suggest that the developmental effects we observe over the prefrontal cortex cannot easily be explained by a straightforward expertise account.

The complexity-dependent developmental changes over prefrontal cortex are somewhat consistent with the predictions of Interactive Specialization (Johnson 2011b), which suggests that there are adjustments over a network of regions as the network accommodates a new function. These adjustments may reflect in part a decoupling of frontal regions away from the default-mode network during development (Fair et al. 2009). In particular, Fair et al. (2007) showed that the frontoparietal and cingulo-opercular networks are less differentiated in the school years, and that both these networks are bridged by the anterior prefrontal cortex and DLPFC regions. In addition, connections between the right frontal cortex and the precuneus appear to weaken, whereas those between the right frontal cortex and the anterior insula/frontal operculum strengthen (Fair et al. 2009). Indeed, it is in right frontal cortex that we see the most dramatic change from more “default-like” activation in children—with complexity-related *decreases* in activation, that is, silly > easy > hard naming—to the more differentiated adult profile of complexity-related *increases* in activation. These developmental differences may only be apparent in task-based fMRI when complexity is increased, and additional neural resources/networks are recruited to support task performance.

## Summary and Conclusions

In this study, we have demonstrated how child and adult brains deal with the demands of an overt picture-naming task. Despite commonalities in activation, we find that there are developmental differences in the activation of regions associated with higher-level visual processing, in orofacial somatosensory, auditory, and auditory-motor areas that appear largely independent of task-complexity. However, by manipulating task complexity, we have been able to interpret developmental differences more comprehensively and explore changes in activation in relation to naming difficulty. We find that bilateral prefrontal regions in adults and children yield fundamentally different patterns in response to naming difficulty. This complexity-dependent difference is in contrast to other regions such as the anterior insula where children and adults modulate activation in a similar fashion. These differences likely reflect adults’ greater language repertoire, differential cognitive demands and strategies during word retrieval and production as well as developmental changes in brain structure. Our findings therefore offer novel insights into how school-age brains cope with naming difficulty within a simple, everyday task that makes no meta-linguistic or literacy demands.

## Supplementary Material

Supplementary material can be found at: http://www.cercor.oxfordjournals.org/

## Funding

Medical Research Council Grant [G0400341] and the Waterloo Foundation. Funding to pay the Open Access publication charges for this article was provided by the Medical Research Council via a block grant to Birkbeck, University of London.

## Supplementary Material

Supplementary Data
