# Supplementary material for: Convergent and Divergent fMRI Responses in Children and Adults to Increasing Language Production Demands
Source: Cereb Cortex. 2014 Jun 6;25(10):3261–77. doi: 10.1093/cercor/bhu120 (PMC4585486; doi:10.1093/cercor/bhu120)
Supplement: Supplementary Data [file supp_25_10_3261__index.html]

Convergent and Divergent fMRI Responses in Children and Adults to Increasing Language Production Demands — Convergent and Divergent fMRI Responses in Children and Adults to Increasing Language Production Demands — Supplementary Data 

# Convergent and Divergent fMRI Responses in Children and Adults to Increasing Language Production Demands

## Supplementary Data

Supplementary Data

**Files in this Data Supplement:**

- Supplementary Figure - doc file
- Supplementary Table - doc file
