# Supplementary material for: Convergent and Divergent fMRI Responses in Children and Adults to Increasing Language Production Demands
Source: Cereb Cortex. 2014 Jun 6;25(10):3261–77. doi: 10.1093/cercor/bhu120 (PMC4585486; doi:10.1093/cercor/bhu120)
Supplement: Supplementary Data [file supp_bhu120_bhu120supp_fig.doc]

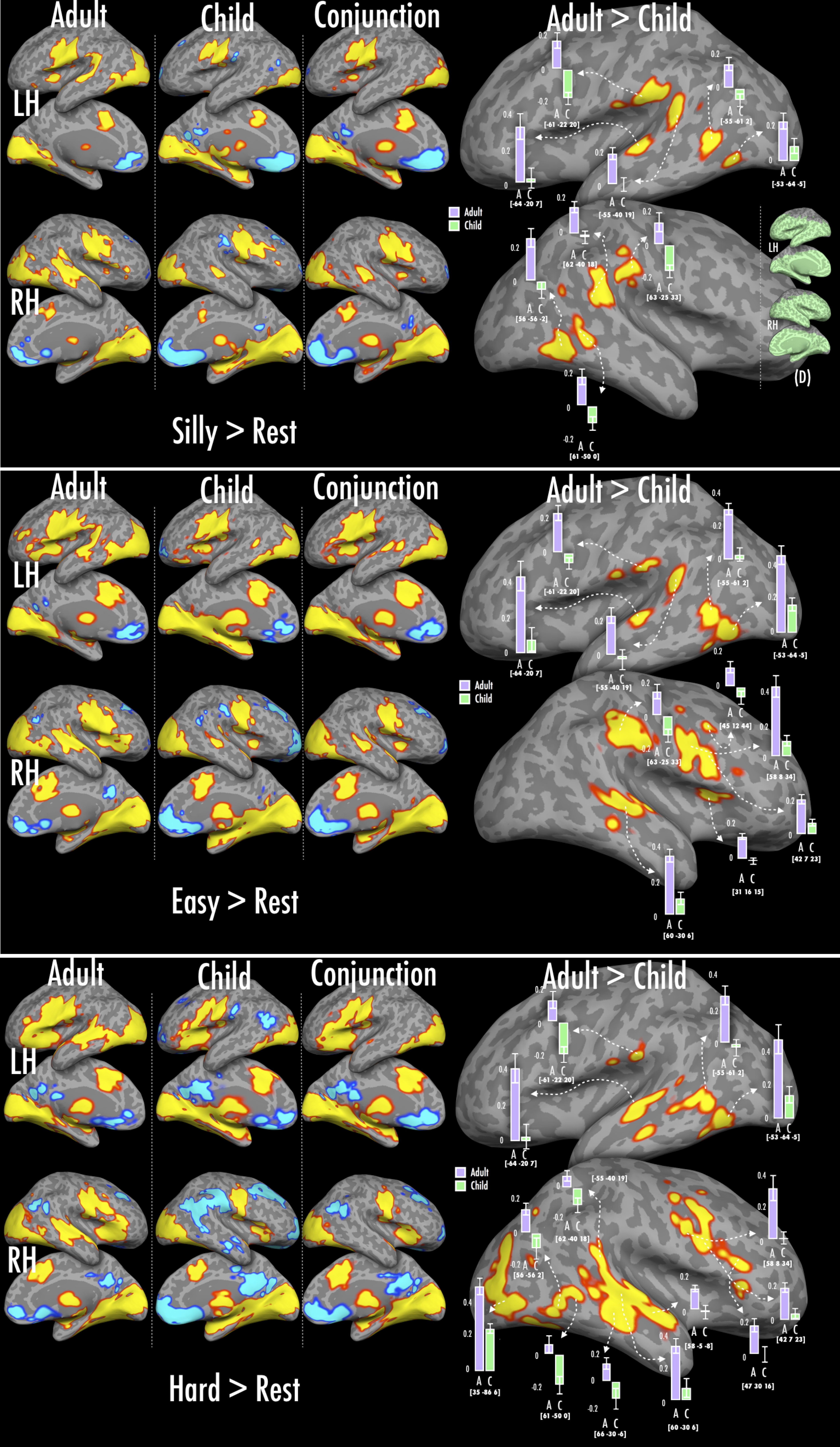


Supplementary Figure 1. Effects of age group (Adults/ Children) on naming ‘silly’ pictures relative to rest (first panel), on naming ‘easy’ pictures relative to rest (second panel) and naming ‘hard’ pictures relative to rest (third panel). (D) depicts areas over which we had coverage in green. In silly > rest, adults show more activation over bilateral posterior middle and inferior temporal gyri, the parietal operculum and the lateral superior temporal gyrus. Similar group differences are observed in easy > rest, in addition, adults show more activation over right anterior supramarginal gyrus, precentral gyrus and inferior frontal gyrus. In hard > rest, adults also show greater activation over the right lateral occipital cortex and middle temporal gyrus, but not over the right anterior supramarginal gyrus. As before, bar graphs show % signal change in a 4mm-sphere centred on the voxel, bars are separated by group; error bars show +/- 1 standard error.


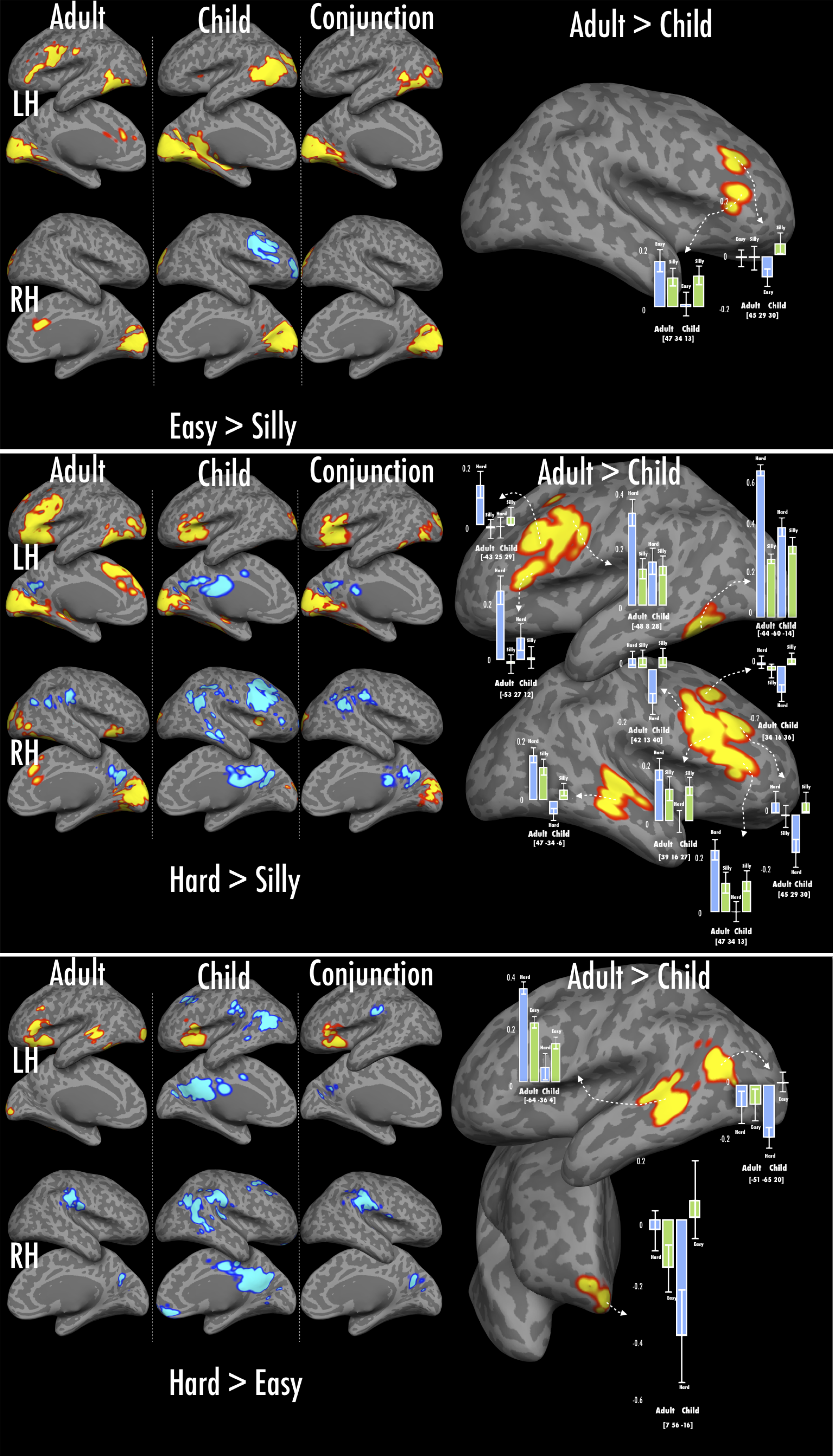


Supplementary Figure 2. Effects of age group (Adults/ Children) on activation for naming easy compared to ‘silly’ pictures (first panel), naming hard relative to silly pictures (second panel) and naming hard compared to easy pictures (third panel). In the easy > silly contrast, adults show a greater positive difference in activation in the right inferior and middle frontal gyri. In the hard > silly comparison, adults show a greater positive difference in activation in bilateral middle and inferior frontal gyrus, right superior temporal gyrus and left inferior temporal gyrus. For hard pictures relative to easy pictures, adults show a greater positive difference in activation in left angular gyrus, the posterior part of the left superior temporal sulcus and the right frontal pole.

Note: in this rendering of activation onto the cortical surface, activations in easy > silly over the right inferior and middle frontal gyri that are contiguous in the volume are non-contiguous on the surface - this is primarily due to voxel-based smoothing over the banks of a sulcus.
