# Supplementary material for: Convergent and Divergent fMRI Responses in Children and Adults to Increasing Language Production Demands
Source: Cereb Cortex. 2014 Jun 6;25(10):3261–77. doi: 10.1093/cercor/bhu120 (PMC4585486; doi:10.1093/cercor/bhu120)
Supplement: Supplementary Data [file supp_bhu120_bhu120supp_table.doc]

*Table S1.* Effects of age group (Adults/ Children) *on percent signal change over the orbital, triangular and opercular parts of the left inferior frontal gyrus. Masks were extracted from an adult’s cortical surface in Freesurfer (normalized to the MNI-152 template) and no overlap between masks was permitted. The values in bold are significant corrected for 18 comparisons.*

| Condition | IFG region | Adult | Adult S.D. | Child | Child S.D. | t | Benjamini-Hochberg corrected p-value |
| --- | --- | --- | --- | --- | --- | --- | --- |
| Silly | Orbital | 0.04 | 0.18 | 0.10 | 0.33 | 0.70 | 0.59 |
|  | Triangular | 0.00 | 0.16 | 0.07 | 0.25 | 1.03 | 0.47 |
|  | Opercular | 0.12 | 0.12 | 0.07 | 0.18 | 0.93 | 0.47 |
| Easy | **Orbital** | 0.14 | 0.19 | 0.15 | 0.30 | 0.14 | 0.90 |
|  | **Triangular** | 0.12 | 0.15 | 0.08 | 0.27 | 0.52 | 0.69 |
|  | **Opercular** | 0.21 | 0.11 | 0.10 | 0.18 | 2.40 | 0.14 |
| Hard | **Orbital** | 0.29 | 0.20 | 0.17 | 0.33 | 1.39 | 0.28 |
|  | **Triangular** | 0.26 | 0.23 | 0.14 | 0.31 | 1.54 | 0.27 |
|  | **Opercular** | 0.25 | 0.13 | 0.14 | 0.22 | 1.88 | 0.19 |
| Easy vs. Silly | **Orbital** | 0.10 | 0.16 | 0.05 | 0.22 | 0.95 | 0.47 |
|  | **Triangular** | 0.11 | 0.11 | 0.01 | 0.18 | 2.16 | 0.15 |
|  | **Opercular** | 0.09 | 0.10 | 0.03 | 0.16 | 1.54 | 0.27 |
| Hard vs. Silly | **Orbital** | 0.25 | 0.17 | 0.06 | 0.35 | 2.26 | 0.14 |
|  | **Triangular** | 0.26 | 0.22 | 0.06 | 0.19 | 3.61 | **0.01** |
|  | **Opercular** | 0.13 | 0.14 | 0.06 | 0.16 | 1.75 | 0.21 |
| Hard vs. Easy | **Orbital** | 0.14 | 0.14 | 0.02 | 0.24 | 2.12 | 0.15 |
|  | **Triangular** | 0.15 | 0.15 | 0.05 | 0.14 | 2.45 | 0.14 |
|  | **Opercular** | 0.04 | 0.06 | 0.03 | 0.12 | 0.25 | 0.85 |

*Table S2. Age-related changes between 7-13 years in percent signal change over the orbital, triangular and opercular parts of the left inferior frontal gyrus. Masks were extracted from an adult’s cortical surface in Freesurfer (normalized to the MNI-152 template) and no overlap between masks was permitted. The values in bold are significant corrected for 18 comparisons.*

| Condition | IFG region | Spearman’s rho | Benjamini-Hochberg corrected p-value |
| --- | --- | --- | --- |
| Silly | Orbital | -0.416 | ***0.05*** |
|  | Triangular | -0.441 | ***0.05*** |
|  | Opercular | -0.331 | 0.14 |
| Easy | **Orbital** | -0.241 | 0.28 |
|  | **Triangular** | -0.204 | 0.37 |
|  | **Opercular** | 0.018 | 0.97 |
| Hard | **Orbital** | -0.002 | 0.99 |
|  | **Triangular** | -0.137 | 0.54 |
|  | **Opercular** | -0.046 | 0.89 |
| Easy vs. Silly | **Orbital** | 0.427 | 0.05 |
|  | **Triangular** | 0.305 | 0.15 |
|  | **Opercular** | 0.276 | 0.2 |
| Hard vs. Silly | **Orbital** | 0.479 | 0.05 |
|  | **Triangular** | 0.352 | 0.12 |
|  | **Opercular** | 0.196 | 0.37 |
| Hard vs. Easy | **Orbital** | 0.310 | 0.15 |
|  | **Triangular** | 0.073 | 0.81 |
|  | **Opercular** | -0.147 | 0.54 |
